# Supplementary material for: Patterns of Intron Gain and Loss in Fungi
Source: PLoS Biol. 2004 Nov 30;2(12):e422. doi: 10.1371/journal.pbio.0020422 (PMC532390; doi:10.1371/journal.pbio.0020422)
Supplement: Table S1 — Also available at http://genes.mit.edu/NielsenEtAl/. (4.3 MB ZIP). [file pbio.0020422.st001.zip › NielsenEtAl/html/914.html]

AN1384.1.NCU04316.1.MG00775.1.FG10145.1


```
 CLUSTAL W (1.82) Multiple Sequence Alignments - Introns Inserted


Sequence 1: NCU04316.1	659 aa
Sequence 2: MG00775.1	630 aa
Sequence 3: FG10145.1	619 aa
Sequence 4: AN1384.1	812 aa
Alignment Length: 851 aa
Number Identitical Residues: 271 aa
Alignment Score (without introns) 14294


MG00775.1 	------------------------------------------------------------
NCU04316.1	-------------------------------------------------------MAATT
FG10145.1 	-------------------------------------------------------MALMS
AN1384.1  	MSGRTPVTIVVEHLDPELGTWSALEYGCIARESHAAGSRFLLSSVPKSLQMPDDLAATKG
          	 :. :. :   .  ... .: :: . .. : .: ::.:    :: ..: . ...  :   

MG00775.1 	------------MRRASTFALLALSWSAAVSAAS--------------~-----------
NCU04316.1	TTTNAGRSMASWKRLSTLIAAFTLSWTSSFVAAA--------------~-----------
FG10145.1 	MIPSPRR--CVVLLSLSLTPTLVAAVNPATSTKA--------------~-----------
AN1384.1  	LEVEHRSVEEVFSDRQSKICLLDPSAKVELSPADGDTFEVFLFGGILD1RTSELRKKGYE
          	   .            :    :  : .    .  ..: .    ..  .  ::.  ... .

MG00775.1 	--------------------------~---------------------------------
NCU04316.1	--------------------------~---------------------------------
FG10145.1 	--------------------------~---------------------------------
AN1384.1  	GRRLGPVQMTTDTAVRVTRMVVHDRV1PLEEIQYIDNPEIVINEHERTEMPFRYVKDSKG
          	.   .. . ::.::   :     .   . .. .  ....   .. . :. .    ..:..

MG00775.1 	------~-------------~------------------~---SAGDYFVRSLPGAPP-G
NCU04316.1	------~-------------~------------------~---GSADYFVHDLPGAPD-G
FG10145.1 	------~-------------~------------------~---AAADYYVRDLPGLPADG
AN1384.1  	KPIMPE0GMVELIKKDAEKT1SSLLSLKCGKPTDPDIPF1LAQSAADYYVESLPGAPE-G
          	..  .. .  .  ...:..: ::  : .....:... .   :..:.**:*..*** *  *

MG00775.1 	PLVKMHAG2HIEVSPEKNGNLFFWHFQNKHIANRQRTVIWLNGGPGCSSEDGALMEVGPY
NCU04316.1	PLVKMHAG2HIEVTPDNNGNLFFWHFQNKHIANKQRTVIWLNGGPGCSSEDGALMEIGPY
FG10145.1 	PKVNMHAG2HIEVTPESHGNLFFWHFENQHIADKQRTVIWINGGPGCSSEDGSMMEIGPY
AN1384.1  	PLLKMHAG2HIEVDPEHNGHLFFWHYQNRHIANRQRTIIWLNGGPGCSSMDGALMEIGPY
          	* ::**** **** *: :*:*****::*:***::***:**:******** **::**:***

MG00775.1 	RLKDDHTLVPNEGSWHEFANLMFVDNPVGTGFSYVNTDSYVTELDEMADQFVIFLEKFFE
NCU04316.1	RLKDENTLVYNDGAWNEFANVLFVDNPVGTGFSYVDTNAYIHELTEMAANFVTFLERWFA
FG10145.1 	RLTDQDNLVYNNGSWNEFANLLFVDNPVGTGFSSVDTNSYIHELKEMADQFVIFLEKWFA
AN1384.1  	RLKDNETLEYNEGSWDEFANLLFVDQPVGTGFSFANTNSYLHELDEMAAQFITFLEKWFA
          	**.*:..*  *:*:*.****::***:******* .:*::*: ** *** :*: ***::* 

MG00775.1 	LFPEYSQDD0IYIAGESFAGQHIPYIAKHILDRNKNS----MTKIKWNLKGLLIGNGWIA
NCU04316.1	LFPEYEHDD0LYIAGESYAGQHIPYIAQAILERNKNAG---PVNRKWNLSGLLIGNGWVS
FG10145.1 	LFPQYDRDD0IYIAGESYAGQHIPYIARAILDRNKKD-----SKTAWNLKGLLIGNGWIS
AN1384.1  	VFPEYERDD0IYIAGESYAGQYIPYIAKAIQDRNKDIHEKQSSSARWNLRGLLIGNGWIS
          	:**:*.:** :******:***:*****: * :***.  ...  .  *** ********::

MG00775.1 	PNEQYRAYLDFSYSKGLLDKNSETAKTLEAQHKDCAKEWEDNGPKVDVAKCESVLQTLLK
NCU04316.1	PKEQYDAYLQFAYEKDIVKKGTDLANKLEIQQRICQKEIAVKPDKIDYPECEAILQDMLQ
FG10145.1 	PAEQYPAYITFGIEKGIIEKDSDNHKKLQADLRSCEKMMASDVGHVDYGECEAILSNMLK
AN1384.1  	PAEQYPAYLSFAYEEGLVEEGSKLGKELETLLSICKSKMETG-PKISITDCEAVLNKLLD
          	* *** **: *. .:.::.:.:.  : *:     * .       ::.  .**::*. :*.

MG00775.1 	LSS-KVEADGKRHCVNMYDVRLRDTYPSCGMNWPPDLVNVTPYLRRKDVVEALHVNP-NK
NCU04316.1	LTAGGVGASGKNQCYNMYDVRLKDDYPSCGMAWPPDLKSVTPYLRKKEVIKALNIND-NK
FG10145.1 	LTK---KGDGDDACINMYDVRLKDSYPSCGMNWPPDLVHLTPYLRKPEVTSALHVDAIKK
AN1384.1  	KTV-----DSNNQCINMYDIRLRDG--SCGTTWPPDLVDVKPYLHTYEVIQALNISP-EK
          	 :      ...  * ****:**:*   ***  *****  :.***:  :* .**::.  :*

MG00775.1 	ATGWTECTGAVGQSFKAQKSKPSIDLLPKILEE-VPILLFSGAEDLICNHIGTEAFIGKM
NCU04316.1	STGWTECNGQVGMNFNP-KTKPSITLLPDILSAGVPILLFSGAEDLICNHLGTEALISNM
FG10145.1 	SVGWTECNGAVGGAFNAKNSKPSVELLPNLLKE-VPIMLFSGAEDLICNHVGTENMINKM
AN1384.1  	ESGWDECDGNVGAAFRPQKSEPSVKLLPGLLESGIEILLFSGDKDLICNHVGTEQLISNM
          	  ** ** * **  *...:::**: *** :*. .: *:**** :******:*** :*.:*

MG00775.1 	TWNGGKGFEVTPGTWAPRRDWTFEGKDAGFWQEARNLTYVLFKDSSHMVPFDFPRRSRDM
NCU04316.1	EWNGGKGFELTPGTWAPRRDWTFEGEPAGFWQQARNLTYVLFYNSSHMVPFDYPRRTRDM
FG10145.1 	EWNGGKGFEVTPGNWAPRRDWDFEGETAGFWQEARNLTYVLVYNSSHMVPFDLPRRSRDM
AN1384.1  	KWAGGTGFETSPGVWAPRHDWTFEDEPAGYYQYARNLTYVLLYNASHMAPFDLPRRTRDM
          	 * **.*** :** ****:** **.: **::* ********. ::***.*** ***:***

MG00775.1 	LDRFMGVDISSIGGNPTDSRLDGEKLPETTVGGAAGNSTSKQEEEK--KKLDEAKWYAYR
NCU04316.1	LDRFMGVDISSIGGQPTDSRLDGEKLPETTVGGAAGNSTSNQAAEK--AKLEMAKWEAYR
FG10145.1 	LDRFMGVDISSVGGDPADSRIDGEKGPKTTVG-SAKNSTQNAAEDEHQKQLDEAKWAAYY
AN1384.1  	VDRFMHVDIASIGGAPADSRIDGEPLPQTSVG-GQPNSTVHQEEEQ--QKIKETEWNAYA
          	:**** ***:*:** *:***:***  *:*:** .  *** :   ::   ::. ::* ** 

MG00775.1 	KSGEVVLVIVAVAAVAWGWYVWRDRRRRRGYQGIFGGSPSE--STTRLPGLGSRIS----
NCU04316.1	KSGELVLVIVIVAAGVWGWFVWKERRKTAG-QGYMGVATGERHSISNNPGPRGNLSGGGD
FG10145.1 	KSGEIVLVIVVIGAVAWGYWIWRERRKGAAYSVLADHDLSR-------------------
AN1384.1  	KSGEAVLVVVIIGVLVWGFLIWRSRRRHNGYSGIAIKSPSR-------------------
          	**** ***:* :.. .**: :*:.**:  . .       ..                   

MG00775.1 	-STPSGLEGFRSKRQNGRDVEAGDFDESELDDLHTQTP----TDARYSVGADSDDEEDAS
NCU04316.1	RTRGQGLAGFRNKRSGRRDVEAQDFDESELDDLHLSKPEDPHADSRYSIGGASDDEEEQK
FG10145.1 	-----RSTGHRAKSQPG-DLETAAFDESELDDLHVTTPG-TSTSARFPKNQDGSEKFVTK
AN1384.1  	---TSIMGRFHNNHSNGADVEAGDFDEAELDDLHSPGLD----RENYAVGEDSDDEAHRQ
          	    .    .: : .   *:*:  ***:******           .:. .  ..::   .

MG00775.1 	-GSRKEGGPSGGRGR---
NCU04316.1	PGKGSSSRQPGGRS----
FG10145.1 	YAE---------------
AN1384.1  	DLPRAQESSSKSENNLVT
          	     .   . . .   :
```
